# Surgical Sutures Filled with Adipose-Derived Stem Cells Promote Wound Healing

**DOI:** 10.1371/journal.pone.0091169

**Published:** 2014-03-13

**Authors:** Ann Katharin Reckhenrich, Bianca Manuela Kirsch, Elizabeth Ann Wahl, Thilo Ludwig Schenck, Farid Rezaeian, Yves Harder, Peter Foehr, Hans-Günther Machens, José Tomás Egaña

**Affiliations:** 1 Department of Plastic Surgery and Hand Surgery, Klinikum rechts der Isar, Technische Universität München, Munich, Germany; 2 FONDAP Center for Genome Regulation, Facultad de Ciencias, Universidad de Chile, Santiago, Chile; 3 Chair for Orthopaedics and Sport Orthopaedics, Klinikum rechts der Isar, Technische Universität München, Munich, Germany; University of California, Merced, United States of America

## Abstract

Delayed wound healing and scar formation are among the most frequent complications after surgical interventions. Although biodegradable surgical sutures present an excellent drug delivery opportunity, their primary function is tissue fixation. Mesenchymal stem cells (MSC) act as trophic mediators and are successful in activating biomaterials. Here biodegradable sutures were filled with adipose-derived mesenchymal stem cells (ASC) to provide a pro-regenerative environment at the injured site. Results showed that after filling, ASCs attach to the suture material, distribute equally throughout the filaments, and remain viable in the suture. Among a broad panel of cytokines, cell-filled sutures constantly release vascular endothelial growth factor to supernatants. Such conditioned media was evaluated in an *in vitro* wound healing assay and showed a significant decrease in the open wound area compared to controls. After suturing in an *ex vivo* wound model, cells remained in the suture and maintained their metabolic activity. Furthermore, cell-filled sutures can be cryopreserved without losing their viability. This study presents an innovative approach to equip surgical sutures with pro-regenerative features and allows the treatment and fixation of wounds in one step, therefore representing a promising tool to promote wound healing after injury.

## Introduction

Insufficient wound healing after tissue injury is associated with an increased risk of infection, loss of tissue functionality, and scar formation, thus generating patient discomfort and elevating treatment expenses. In many cases, currently available therapeutic options are not satisfying, generating a tremendous demand for alternative strategies to treat problematic wounds.

After tissue injury, wound healing occurs in three dynamic phases: inflammation, proliferation, and remodeling, which are orchestrated by auto- and paracrine mechanisms. Although a multitude of cell types are involved in these processes, it is highly recognized that mesenchymal stem cells (MSCs) play a key role in the promotion of wound healing [1]–[4]. MSCs can differentiate into cells from different lineages and, therefore, have been utilized to rebuild tissues in several tissue engineering approaches [5], [6]. Current perspectives also highlight their ability to secrete regulatory molecules that act either directly by auto- or paracrine signaling or indirectly as trophic mediators. This capacity enables MSCs to create a regenerative microenvironment at sites of tissue damage by fostering key processes in wound healing such as immunosuppression, cell homing, and migration [7]–[10]. The combined use of MSCs and biomaterials is promising for tissue engineering and regeneration and has been successfully used in pre- and clinical trials, describing accelerated healing of human cutaneous wounds after application of an MSC-loaded fibrin spray [11].

Tissue defects often require mechanical fixation with surgical sutures. Since sutures are in direct contact with the wound, they represent an excellent opportunity for local delivery of active molecules or cells and, therefore, improve wound healing. The potential use of the suture itself as a carrier system presents an emerging field of research, thus innovative approaches have been recently reported. The coating of sutures with antibiotics has shown to be effective against local infections and, therefore, is already available for clinical applications [12], [13]. Moreover, the use of sutures coated with bioactive molecules, such as insulin-like growth factor-1 or growth differentiation factor-5 are able to promote healing in rat models of anastomoses [14] or tendon repair [15], respectively. However it has been shown that suture coatings lead to physical disruption of the bioactive reagent during the mechanically bearing suturing process [16]. Therefore, new strategies are required to prevent these shortcomings.

Adipose tissue represents an easily accessible and abundant source of MSCs, so called adipose-derived stem cells (ASC). Aiming to develop a bioactive suture to promote wound healing and improve scaring, in this work we combine the use of ASCs and surgical sutures to locally deliver pro-regenerative factors directly to the wound. Here we present a technology to seed ASCs homogenously into the polyfil suture, thus preventing physical disruption during suturing. Moreover, basic mechanisms of action, like auto- and paracrine signaling of ASCs in the suture are also revealed in this work.

## Materials and Methods

### Ethics statement

The ethics committee of the Medizinische Fakultät at the Technische Universität München has approved all research involving human participants and all patients provided their written consent to participate in this study.

### Isolation of adipose-derived stem cells (ASC)

ASCs were isolated from human tissues. Here 8 tissue samples were obtained from 7 female and 1 male donor ranging from 24 to 67 years old (46.12±15.25 yrs; average ± SD). Samples were taken from different body areas including abdomen, flanks and legs. Smokers were discarded from this study.

Samples were digested using sterile filtered 0.1% collagenase A solution (Roche, Grenzach-Wyhlen, Germany) in phosphate buffered saline (PBS, Biochrom, Berlin, Germany) incubated at 37°C until a fat emulsion emerged while inverting every 10 minutes. Culture medium (Dulbecco's Modified Eagle Medium (DMEM), 10% fetal calf serum (FCS), 10 U/ml penicillin, 10 mg/ml streptomycin sulfate, and 25 μg/ml amphotericin B) was added to stop collagenase digestion, mixed gently, and centrifuged (1300 rpm, 10 minutes, 20°C). After centrifugation the supernatant was aspirated and the pellet was suspended in 5 ml PBS and filtered through a 100 μm pore size cell strainer. Then, the sample was centrifuged again as before and the supernatant was discarded. The pellet was suspended in culture medium, transferred to 175 cm^2^ culture flasks (Greiner Bio-One, Frickenhausen, Germany) and cultivated at 37°C and 5% CO_2_. The medium was replaced the following day to remove non-adherent cells. Afterwards the medium was replaced once a week. Cells were passaged after reaching 80-90% confluence.

### Characterization of ASCs

Cells (2.5×10^4^) were seeded in a 24 well plate and cultivated at 37°C and 5% CO_2_. To examine the cell morphology, cells were fixed after 24 hours, stained for 1.5 hours with phalloidin (Sigma Aldrich, Deisenhofen, Germany) according to the manufacturer's instructions, and mounted in ProLong Gold Antifade Reagent with DAPI (Invitrogen, Oregon, USA). To determine multipotency of the cells, chondrogenic, adipogenic, and osteogenic differentiation experiments were performed.

#### Chondrogenic differentiation

Chondrogenic differentiation of ASC was performed according to the Poietics human ADSCs-chondrogenesis protocol (Lonza, Basel, Switzerland). Control cells were incubated with culture medium during the same period. Chondrogenic pellets were harvested after 21 days and fixed in 3.7% formaldehyde (Fischar, Saarbrücken, Germany) for 10 minutes. After washing with sterile PBS, cells were fixed in 3.7% formaldehyde followed by a staining for 30 minutes with 1% Alcian blue in 3% acetic acid (Roth, Karlsruhe, Germany). Then pellets were washed with tap water and analyzed with a Stemi 2000-CS microscope (Zeiss, Jena, Germany).

#### Adipogenic differentiation

ASCs (2×10^5^) were seeded in 24 well plates in 1 ml culture medium and incubated at 37°C and 5% CO_2_ until 100% confluence. Afterwards, culture medium was replaced by supplemented Adipogenesis Induction Medium (AIM, Lonza, Basel, Switzerland) and cells were incubated for 3 days at 37°C and 5% CO_2_. Next, AIM was replaced by supplemented Adipogenic Maintenance Medium (AMM, Lonza, Basel, Switzerland) followed by 3 days of incubation at 37°C and 5% CO_2_. This cycle was repeated 3 times. Control cells were incubated with AMM during the same period. Next, cells were incubated for 7 days with AMM and medium was replaced every 2–3 days. After 20 days, cells were fixed with 3.7% formaldehyde (Fischar, Saarbrücken, Germany) for 1 hour and Oil Red O staining was performed to evaluate lipid vacuole formation. Therefore, formaldehyde was discarded and cells were rinsed three times with PBS (Biochrom, Berlin, Germany). Subsequently, cells were covered with 500 μl of 60% isopropanol for 5 minutes. After removing isopropanol, cells were air-dried and 200 μl of Oil Red O solution (3 mg/ml in 100% isopropanol) was added for 10 minutes. Then staining solution was removed, cells were washed with tap water and analyzed with an Eclipse TE2000-S microscope (Nikon, Germany).

#### Osteogenic differentiation

ASCs (6.2×10^3^) were seeded in 1 ml culture medium in 24 well plates. After 24 hours, culture medium was replaced by Osteogenesis Induction Medium (OIM, Lonza, Basel, Switzerland). OIM was changed every 3–4 days. Control cells were incubated with culture medium during the same period. After 3 weeks, cells were fixed with 3.7% formaldehyde (Fischar, Saarbrücken, Germany) and von Kossa staining was performed according to manufacturer's instructions (von Kossa acc. McGee-Russel, Diapath, Martinengo, Italy). Cells were analyzed with an Eclipse TE2000-S microscope (Nikon, Germany).

### Cell filling of sutures

Biodegradable surgical sutures (Vicryl, Johnson & Johnson, Norderstedt, Germany) were cut into 3 cm pieces. ASCs (5×10^5^) were suspended in 8 μl culture medium and injected with a 100 μl HPLC syringe (Göhler Analysentechnik, Chemnitz, Germany) into the cavity of the 3 cm long suture. Each cell-filled suture was incubated over night at 37°C and 5% CO_2_ in a 75 cm^2^ cell culture flask containing 20 ml culture medium. After 24 hours, sutures were placed into 24 well plates, each in a separate well containing 1 ml culture medium for further cultivation.

### Scanning electron microscopy (SEM)

After 10 days under standard culture conditions, cell-filled sutures were fixed with 3% glutaraldehyde (Sigma Aldrich, Deisenhofen, Germany), dehydrated, and sputter-coated with platinum (Emitech K550, 15 mA, 120 s). SEM was carried out using a S-4800 microscope (Hitachi, Krefeld, Germany) in Hi-Vac mode by applying an acceleration voltage of 5 kV and detecting secondary electrons for imaging.

### Laser scanning microscopy (LSM)

After 10 days under standard culture conditions, cell-filled sutures were fixed with 3.7% formaldehyde (Fischar, Saarbrücken, Germany). In order to analyze the distribution and interaction of cells in the suture, samples were stained for 1.5 hours with phalloidin (Sigma Aldrich, Deisenhofen, Germany) according to manufacturer's instructions. Finally, samples were mounted in ProLong Gold Antifade Reagent with DAPI (Invitrogen, Oregon, USA). Fluorescence confocal z-stacks of DAPI stained sutures were acquired with a LSM 710 microscope (Zeiss, Jena, Germany).

### Quantification of metabolic activity of cells in the suture

At indicated time points after cell seeding, sutures were incubated for 1 hour in culture media containing 500 mg/ml of 3-(4,5-dimethyl-2-thiazolyl)-2,5-diphenyl-2H-tetrazolium bromide (MTT, Sigma Aldrich, Deisenhofen, Germany). Next, MTT solution was removed and sutures were washed with PBS (Biochrom, Berlin, Germany). After, sutures were incubated in 500 μl dimethyl sulfoxide (DMSO, Sigma Aldrich, Deisenhofen, Germany) until all formazan blue was extracted. Finally, absorbance was measured at 560 nm.

### Biomechanical characterization

ASC-filled sutures (1×10^6^ cells/6 cm long suture) were incubated in culture medium for 10 days at 37°C and 5% CO_2_. Non-seeded sutures were incubated under the same conditions as controls. A tensile test was applied using a uniaxial test system (zwicki 1120, Zwick Roell Ulm, Germany) and a 2.5 kN load cell (KAF-Z 2.5 kN, class 0.05%, Angewandte System Technik GmbH, Dresden, Germany). The sutures were clamped on both sides leading to a freely suspended length of 30 mm. To avoid shear forces, sutures were mechanically allowed to move free in the perpendicular plane and in rotation. With a constant rate of 5 mm/min, the sutures were loaded until failure. From the force and displacement sensor channels stiffness, maximum force and the elastic limit were determined. Tests were carried out one after another at a constant temperature of 20°C and the specimens were kept moist at all times.

### Quantification of VEGF and SDF-1α

ASCs (5×10^5^) were filled into 3 cm long sutures as previously described. After 8 days, medium was replaced by culture medium containing 2% FCS. At indicated time points the concentration of vascular endothelial growth factor (VEGF) and stromal derived factor-1α (SDF-1α) in supernatants and protein extracts were measured by ELISA according to manufacturer's instructions (Quantikine human VEGF Immunoassay, Quantikine human CXCL12/SDF-1α Immunoassay, R&D Systems, Minneapolis, USA). The suture samples were homogenized with a Mini-Beadbeater (10 s pulses; Biospec Products, Inc., Bartlesville, USA) in 2 ml screw-cap tubes (VWR Scientific Products, West Chester, USA) containing 1 g (Ø 2.5 mm; Biospec Products, Inc., Oklahoma, USA) and 500 μl of PBS. Samples were kept on ice during the whole procedure. After centrifugation (7600 rpm for 5 minutes at 4°C), 100 μl (SDF-1α ELISA) or 200 μl (VEGF ELISA) of the sample was analyzed.

### Cytokine release from filled sutures

ASC-filled sutures containing 5×10^5^ cells were cultured for 8 days and medium was replaced by culture medium containing only 2% FCS for an additional 48 hours. Next, medium was removed and the presence of 55 different cytokines was evaluated simultaneously using a cytokine protein array (Proteome Profiler Human Angiogenesis Array Kit, R&D Systems, Minneapolis, USA). Culture medium from non-seeded cells was used as a negative control. The cytokine profile was quantified using ImageJ microarray profile software (http://rsbweb.nih.gov/ij/). Results were expressed as the percentage of pixel density relative to the positive control.

### *In vitro* wound healing assay

Conditioned media from the supernatant of cell-filled sutures and was used for cell migration analysis. Here, ASCs (5×10^5^) were filled into sutures as described before and medium was replaced by culture medium containing 2% FCS after 8 days. Conditioned media was collected after additional 48 hours under standard culture conditions. Next, ASCs (1×10^4^) were seeded into a culture-insert (Ibidi, Martinsried, Germany) in 70 μl culture medium. Cells reached confluence after 24 hours and culture inserts were removed. The medium was replaced by conditioned medium or fresh culture media (2% FCS) for controls. After 30 hours, cells were stained with calcein AM (Invitrogen, Oregon, USA), according to manufacturer's instructions, to visualize living cells. Micrographs were taken with a Axio Observer.Z1m microscope (Zeiss, Göttingen, Germany) and cell migration in the open wound area was quantified using TScratch as previously described [17]. Results are expressed as the percentage of open wound area at 30 hours compared to 0 hours.

### Survival of cells after suturing

ASCs (1×10^6^) were filled in the distal 6 cm of a 9 cm long segment of the suture as previously described. The proximal 3 cm of the segment remained empty in order to thread in the eye of a reusable surgical needle (Johnson & Johnson, Norderstedt, Germany) and the seeded sutures were passed intradermally through *ex vivo* human skin obtained from patients who underwent abdominoplasty. After 10 days in standard culture conditions, the metabolic activity of cells in the suture was examined before and after suturing.

### Cryopreservation

ASCs (5×10^5^) were filled into 3 cm long sutures as previously described and cultured for 24 hours under standard culture conditions. Next, cell-filled sutures were frozen in culture medium containing 10% DMSO. After another 24 hours, cell-filled sutures were thawn and cultured in a 24 well plate containing 1 ml culture medium. Medium was exchanged with fresh culture medium on the following day. The metabolic activity was determined 8 days after thawing by MTT analysis. Control sutures were cultured for the same period of time but without freezing.

### Statistical analysis

All assays were repeated in at least 3 independent experiments. Statistical analysis was performed using student's t-test. Data are expressed as mean ± standard error (SEM).

## Results

### Cell isolation and characterization

**Figure 1:**
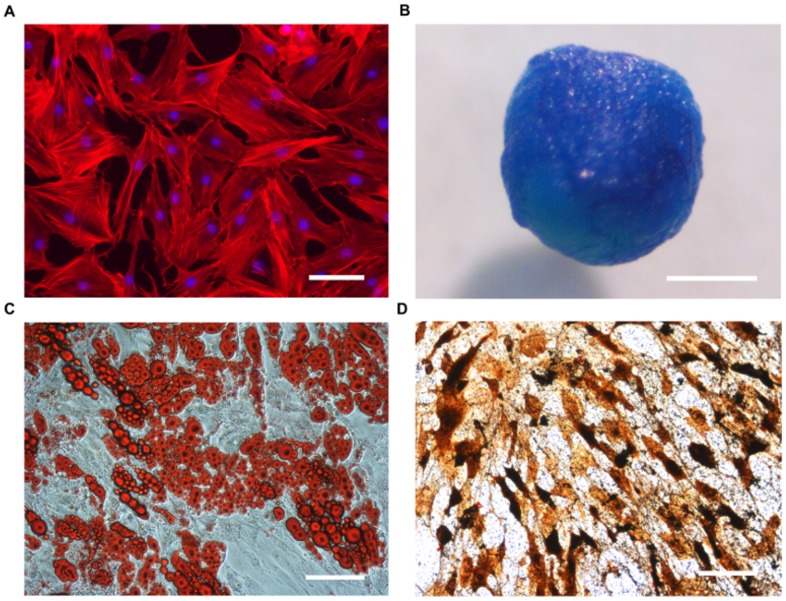
Cell characterization. After isolation, cells adhered to tissue culture plastic showing a fibroblast-like morphology (A, DAPI (blue)/phalloidin (red) staining) and multipotency (B–D). Chondrogenic (B), adipogenic (C) and osteogenic (D) differentiation potential was confirmed by Alcian blue, Oil red O and von Kossa staining, respectively. Scale bars represent 100 μm in A, C, D and 1 mm in B.

ASCs were isolated from human fat tissue and characterized in terms of plastic adherence, morphology, and differentiation potential. Isolated cells adhered to the culture dish, presenting a typical fibroblast-like morphology (Fig. 1A). In order to determine their mesenchymal differentiation capacity, the expression of typical features of cartilage, bone, and fat cells was determined after cultivation under each particular differentiation condition. Results showed that cells were positive for markers of the chondrogenic (Fig. 1B), adipogenic (Fig. 1C), and osteogenic (Fig. 1D) lineage evaluated by Alcian blue, von Kossa, and Oil red O staining, respectively.

### Cell filling of the suture

**Figure 2:**
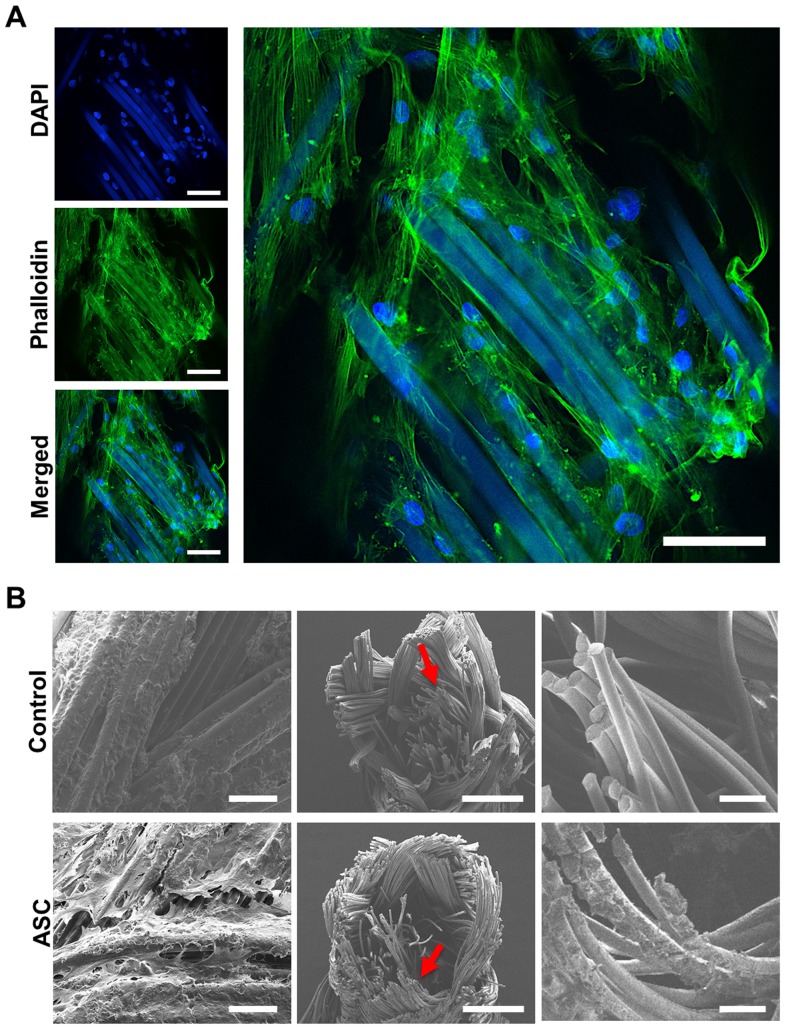
Cellular distribution and attachment in the suture. ASCs were filled into biodegradable sutures. Laser scanning microscopy (LSM, A) shows attached cells (DAPI, blue) along the suture surface interacting with each other (phalloidin, green) and the suture itself (DAPI, blue/phalloidin, green). The suture material is autofluorescent. As observed by scanning electron microscopy (SEM, B), cells were distributed throughout the surface (left) and the inner filaments (middle, right) of the suture. Scale bars represent 50 (A and B left), 500 (B, middle), and 100 μm (B, right).

**Figure 3:**
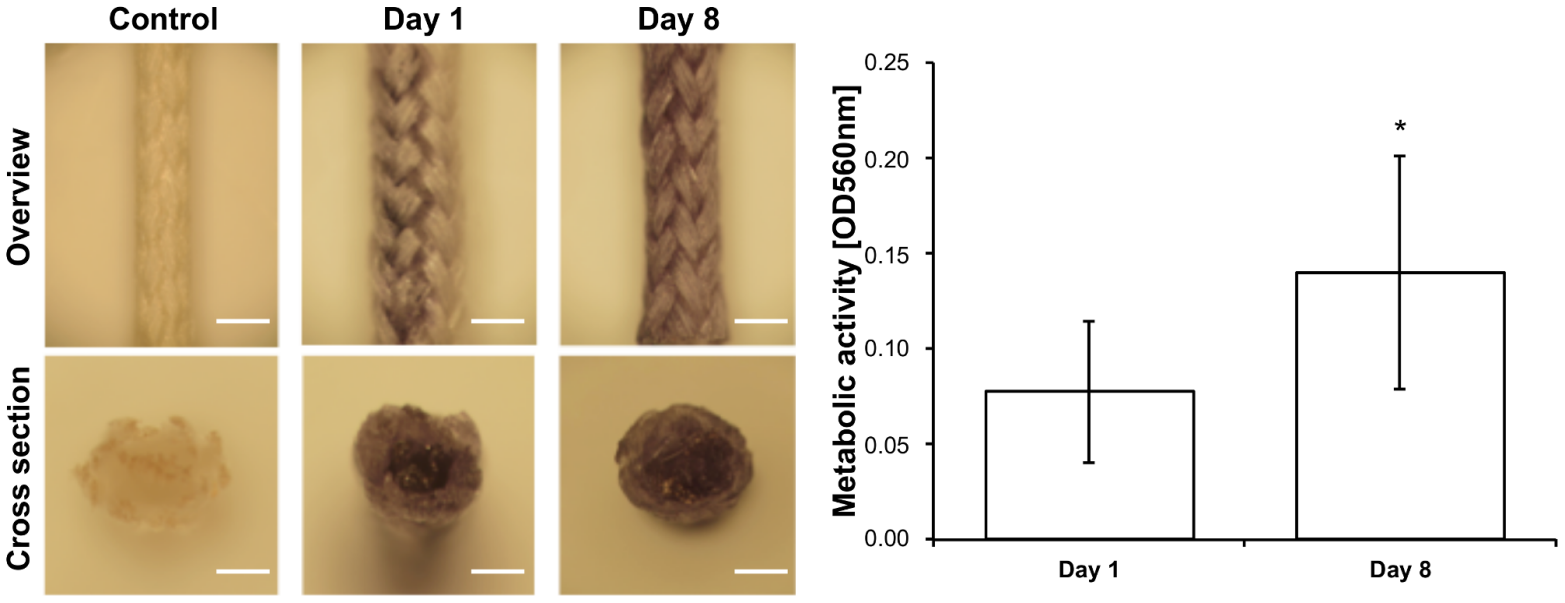
Cell viability and proliferation in the suture. The metabolic activity of ASCs in the suture was determined by MTT assay. Metabolically active cells (dark precipitate) were observed on the surface (overview) and in the inner cavity (cross section) of the suture at day 1 and 8 after seeding. Moreover, a significant increase of metabolic activity was observed from day 1 to 8. Scale bar represents 2 mm. p<0.05.

After isolation, ASCs were filled into biodegradable sutures and their distribution and attachment was studied by LSM and SEM. Results obtained by LSM showed that seeded cells distribute homogeneously in the suture, attaching directly, and enwrapping the filaments (Fig. 2A). A strong intercellular interaction was also observed, thus the seeded ASCs formed a complex tridimensional arrangement in the suture. SEM micrographs confirmed these results and showed a layer of ASCs covering the whole surface of the suture and filaments within the inner cavities (Fig. 2B). A rough, non-homogenous surface was observed in control sutures with SEM, whereas filaments in the inner cavity appeared smooth (Fig. 2B). After showing that cells can be incorporated in the suture material, attach, and distribute equally, the metabolic activity of ASCs after filling in the suture was evaluated by MTT metabolic assay (Fig. 3). A macroscopic analysis showed the formation of formazan blue precipitates throughout the polyfil suture, showing that cells were metabolically active at day 1 and 8 after seeding (Fig. 3). Moreover, a quantitative analysis showed that the metabolic activity at day 8 was significantly increased when compared to day 1 (p<0.05).

### Biomechanics

**Figure 4:**
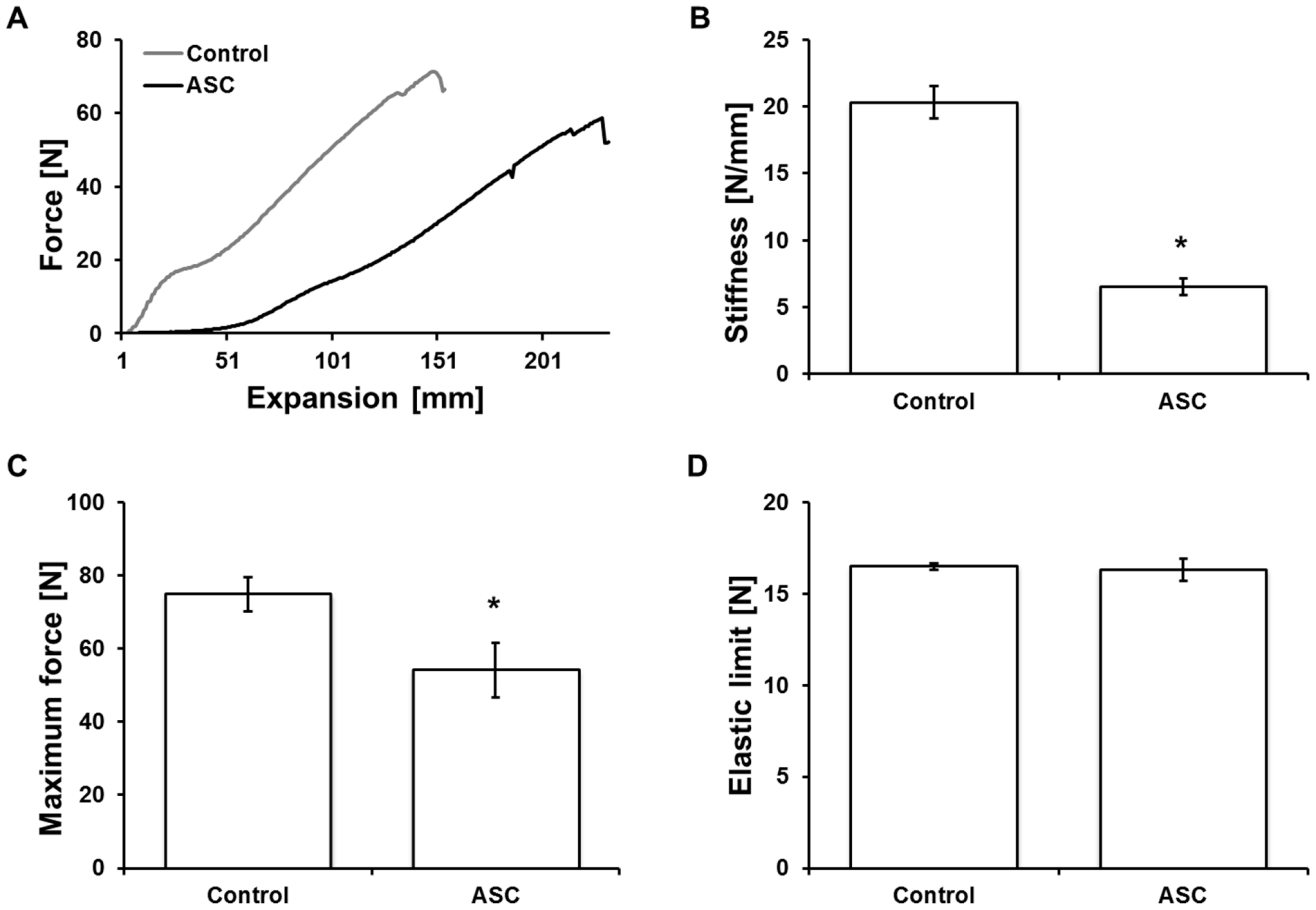
Biomechanical properties of ASC-filled sutures. A force/expansion profile (A) was recorded to evaluate stiffness (B), maximum force (C), and the elastic limit (D) of ASC-filled sutures. Results show that stiffness and maximum force were significantly reduced when compared to cell-free sutures (control), but the elastic limit remained similar. p<0.001.

After determining that cells can be filled and cultured in surgical sutures, the biomechanical properties of the ASC-filled sutures were evaluated by force-expansion profiles. The force-expansion profiles (Fig. 4A) showed significant differences (p<0.001) between control and ASC-filled sutures in stiffness (Fig. 4B) and maximum force (Fig. 4C). In more detail, the stiffness decreased to 32% from 20 N/mm in the control group to 7 N/mm in the cell-filled suture group and the maximum force was reduced to 72% from 75 N to 54 N. In both groups, sutures showed an elastic limit at an equal level of 16 N for the ASC-filled sutures and the control group (Fig. 4D).

### Cytokine release

**Figure 5:**
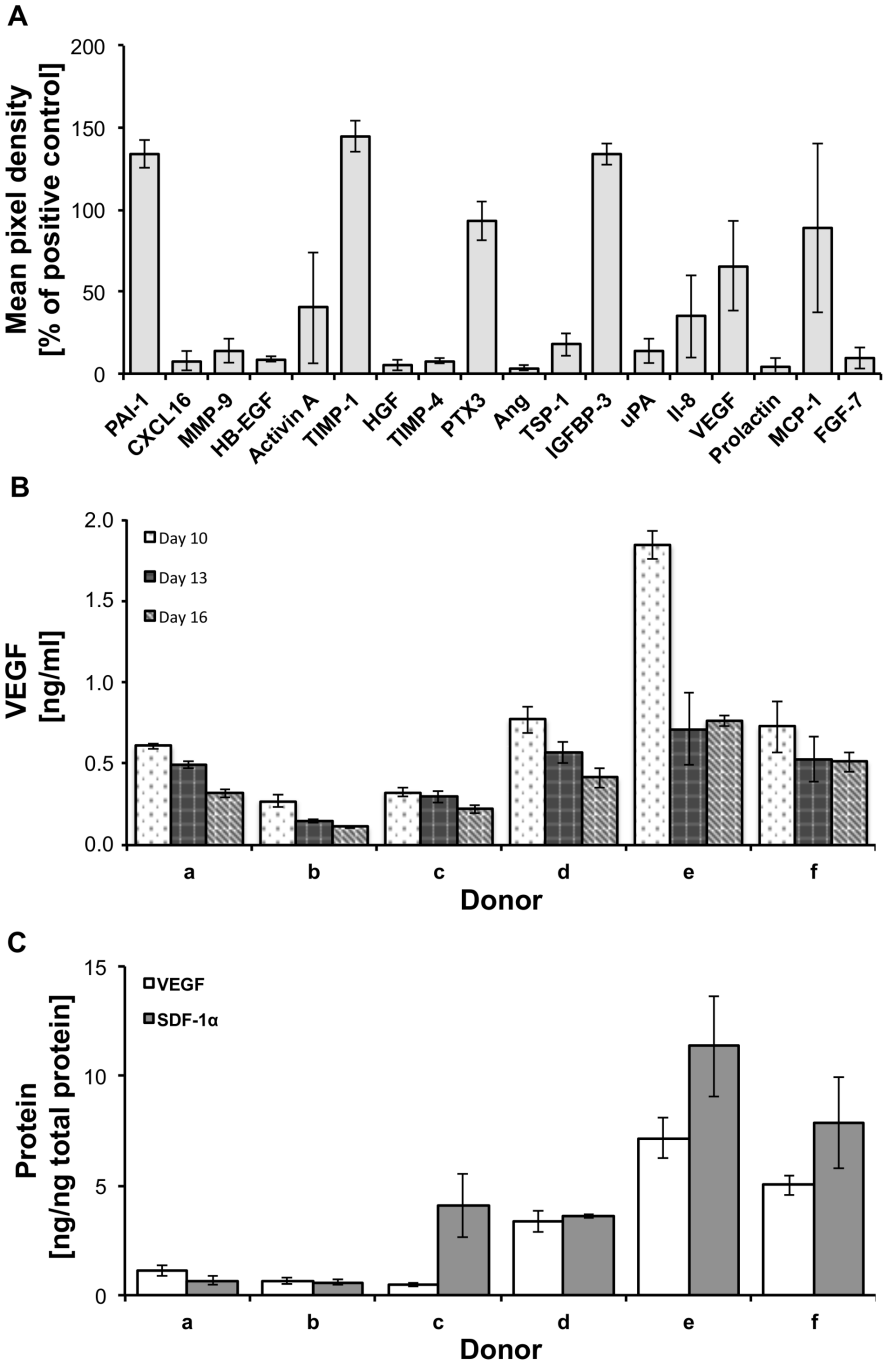
Cytokine release from ASC-filled sutures. A cytokine array detected a multitude of different cytokines released from ASC-filled sutures (A). VEGF was constantly released to cell culture medium for at least 16 days (B). VEGF and SDF-1α were detected in protein extracts of ASC-filled sutures 16 days after seeding (C).

To evaluate the paracrine profile of ASCs in sutures, a cytokine array was performed 10 days after seeding. Among the 55 cytokines analyzed, relevant amounts of the following molecules were detected in the culture media of sutures filled with ASCs obtained from 6 different donors: Plasminogen activator inhibitor-1 (PAI-1), chemokine (C-X-C motif) ligand 16 (CXCL16), matrix metallopeptidase 9 (MMP-9), heparin-binding EGF-like growth factor (HB-EGF), activin A, tissue inhibitor of matrix metalloproteinase-1 (TIMP-1), hepatocyte growth factor (HGF), tissue inhibitor of matrix metalloproteinase-4 (TIMP-4), pentraxin 3 (PTX3), angiogenin (Ang), thrombospondin-1 (TSP-1), insulin-like growth factor-binding protein-3 (IGFBP-3), urokinase plasminogen activator (uPA), interleukin-8 (Il-8), VEGF, prolactin, monocyte chemotactic protein-1 (MCP-1) and fibroblast growth factor-7 (FGF-7) (Fig. 5A). Since secreted factors may vary according to time after seeding and between donors, the concentration of VEGF and SDF-1α was compared in 6 donors at 3 different time points. Results showed that all ASC-filled sutures constantly released VEGF for at least 16 days *in vitro*, showing a general tendency to decrease over time. The decrease was significant in 5 samples (p<0.05). In addition, differences among the donors were observed at different time points (Fig. 5B). Analysis of SDF-1α release presented a different outcome. SDF-1α release was detected only in 1 out of 6 donors (Donor c) and the concentration did not vary over time with mean values of 213, 193, and 203 pg/ml at day 10, 13, and 16, respectively (data not shown). In addition, the accumulation of VEGF and SDF-1α in the suture material was quantified 16 days after seeding. All samples were positive for both molecules and differences among donors of up to 15 times for VEGF and 19 times for SDF-1α were observed (Fig. 5C).

### Biofunctionality

**Figure 6:**
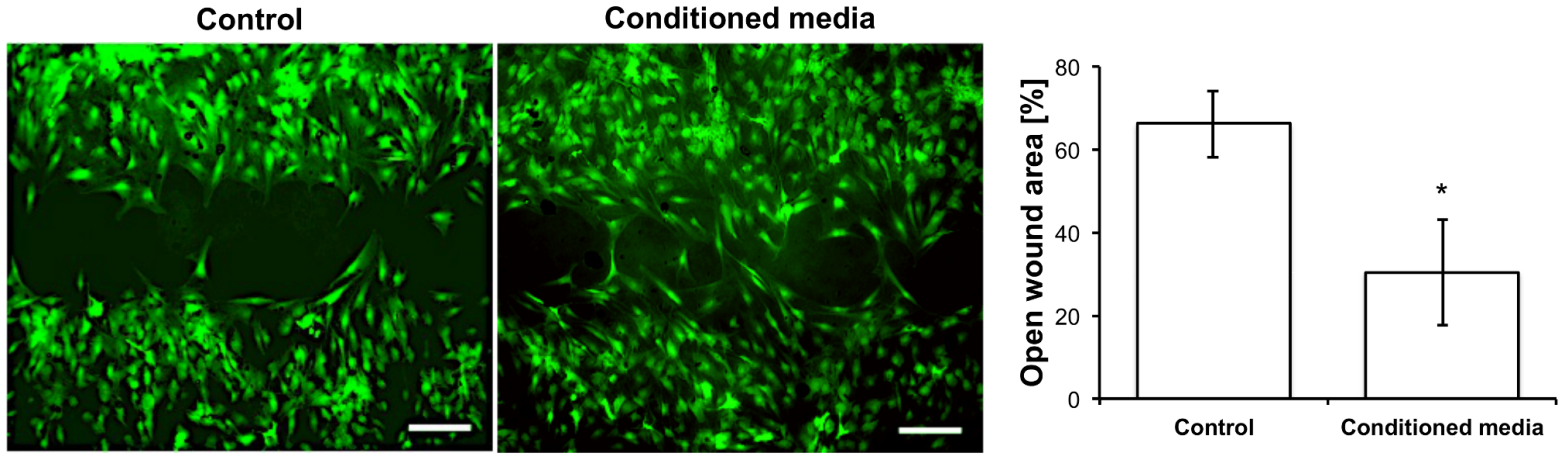
Biofunctionality of ASC-filled sutures. Fresh media (control) and media conditioned with cell-filled sutures (conditioned) were tested in an *in vitro* wound healing assay (scratch assay) with ASCs. After 30 hours, a significant reduction in the open wound was observed when conditioned media was applied. Scale bars represent 500 μm. p<0.05.

An *in vitro* wound healing assay revealed the biofunctionality of the released cytokines (Fig. 6). Therefore, a scratch was performed in a confluent layer of ASCs to create an open wound area. ASCs were cultured with conditioned media from ASC-filled sutures and fresh media (control). Results showed that conditioned media induced a significant decrease in the open wound area after 30 hours of cell migration (Fig. 6).

### Clinical perspective

**Figure 7:**
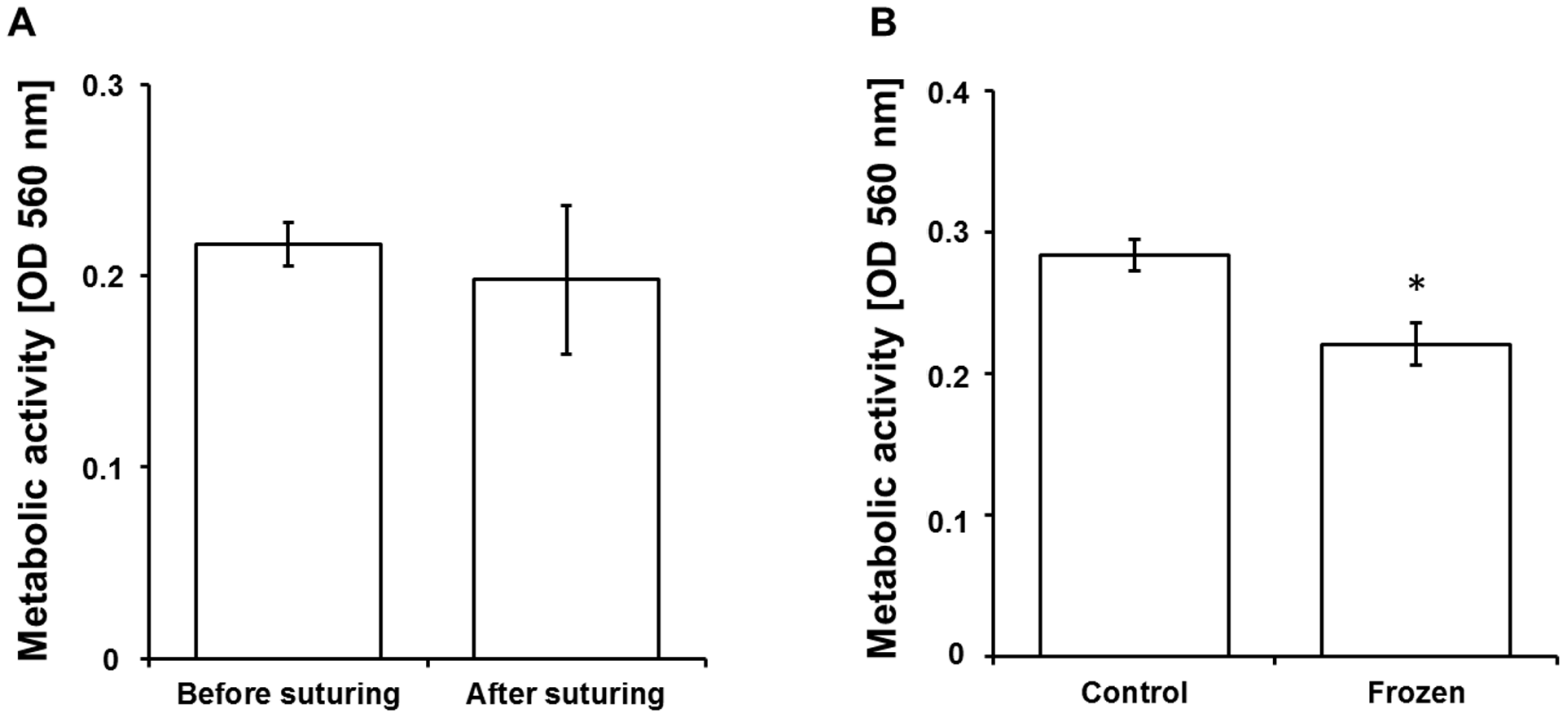
Clinical perspective. To determine the effect of mechanical stress on cell survival during suturing the metabolic activity of cell-filled sutures was determined before and after suturing in an *ex vivo* model using human skin, where no significant difference were observed (A). The potential to produce storable cell-filled sutures was evaluated. Therefore, the metabolic activity of ASCs in the suture was determined after freezing relative to unfrozen sutures. After freezing, 78% of the metabolic activity was preserved (B). p<0.001.

The feasibility of using ASC-filled sutures in clinical settings was evaluated in an *ex vivo* model using human skin samples. Whereby, a full thickness incisional surgical wound was performed and closed by an intradermal suture. An X-ray analysis using a suture made of steel showed that the suture aligns and is in direct contact with the wound margins and therefore is able to deliver ASCs and released cytokines along the wound (data not shown). In order to evaluate the survival of cells in the suture after the mechanical bearing suturing process, cell viability was determined before and after suturing. Results showed no significant decrease in metabolic activity of cells in the suture after suturing compared to control (Fig. 7A). To facilitate the use of ASC-activated sutures in a clinical setting, storable ready to use sutures were generated. Therefore, freezing procedures were standardized and the metabolic activity of ASCs in the suture was determined after freezing. As shown in Fig. 7B, cells were metabolically active after thawing, preserving about 78% of the metabolism.

## Discussion

Wound healing is classically divided in three overlapped phases. First bacteria and debris are removed during the inflammatory phase, followed by the proliferative phase, characterized by the formation of new blood vessels, extracellular matrix deposition, granulation tissue formation, epithelialization, and wound contraction. Finally, in the remodeling phase collagen fibers are reorganized and cells that are no longer needed are removed by apoptosis, thus giving the final shape to the repaired tissue. Interestingly, the paracrine factors released by MSC have shown to be capable of modulating all three phases of wound healing [18]. The beneficial role of MSC in wound healing as anti-inflammatory and immunoregulative agents is described in detail in several reviews [19], [20]. Moreover, the soluble factors released by MSCs have shown to promote cell migration, proliferation, and angiogenesis in the proliferative phase and improve scaring during tissue remodeling [18].

Unsatisfactory therapeutic options in wound healing require alternative approaches for treatment [21]. Most strategies involve the use of surgical sutures which, to date, are only used to fix tissues by mechanical means but do not actively contribute to the healing process. As MSCs have been extensively described to promote wound healing [22] and ongoing clinical trials have shown a great potential on the use of ASCs in regenerative medicine [23], in this work, we present a new approach where surgical sutures are activated by filling them with human ASCs. This key feature allows keeping cells on site and protecting them from the mechanical stress induced by suturing. Interestingly, in our study the incorporation of ASCs into the inner core of the suture did not affect their viability and increased metabolic activity was observed over a period of 8 days, suggesting cell proliferation (Fig. 3). As observed by SEM micrographs, we found a rough surface structure of untreated, native Vicryl in contrast to the smooth inner filaments, which might be the result of a copolymer-coating (polyglactin 370 and calciumstearat) [24]. Although cells were filled into the suture, results show that they were also present on the surface. This shows a certain capacity of the cells to move out to the surface, suggesting that a cell migration from the core of the suture to the surrounding tissues would be possible *in vivo*.

Recent reports have evaluated the possibility of using cell-coated sutures to deliver cells. For instance, it was shown that suture-based cell engraftment in rat heart muscles is more stable than *i.m.* injection [16]. In another work, the authors described that MSC-coated sutures can modulate inflammation *in vivo* [25]. A drawback of these studies is that most cells remain at the initial insertion site and the mechanical stress induced by the suturing itself may strongly affect the viability of the cells. This phenomena is probably also occurring here with the ASCs that migrate to the surface of the suture. However, since the main cell reservoir is in the core, such mortality might not be relevant when compared to coating approaches. In addition, the basic mechanisms of action of such cell-coated sutures have not been described to date.

MSCs have been shown to improve tissue regeneration by direct differentiation [5], [6], as well as by the release of a multitude of cytokines that contribute to create a pro-regenerative microenvironment in the wound area [7]–[10]. Since ASCs located in the core of the suture are protected from mechanical stress, they may represent a constant source to release such paracrine factors. Thus, in this work we also focused on the cytokine profile evaluation of the ASC-filled sutures. It has been extensively shown that ASCs secrete nearly all of the growth factors that take part in normal wound healing, promoting healing by increasing vessel density, granulation tissue thickness, collagen deposition, and also improving the cosmetic appearance of resultant scars [26]. In order to have an overview of the paracrine profile of the seeded ASC, we evaluate the release of 55 cytokines simultaneously, which are part of a commercially available kit. Most probably, the released cytokines represent only a fraction of the total molecules, but show the paracrine potential of the seeded sutures and confirm the presence of at least 18 bioactive molecules which are involved in key processes of wound healing such as immunomodulation, angiogenesis, and tissue remodeling (Fig. 5A). For instance, PTX3, IL-8, and MCP-1 modulate neutrophil and macrophage infiltration during wound repair and, therefore, the inflammatory response. Angiogenesis is mediated by MCP-1 and IL-8. Wound re-epithelialization is triggered by FGF7, HB-EGF, MCP-1, whereas TGF-β1, which was not detected here, delays re-epithelialization and promotes scar formation *in vivo* [27]. PTX3 also regulates inflammation after secretion in response to microbial moieties and inflammatory signals [28]. IL-8 plays a key role in wound inflammation and its topical application has been shown to increase re-epithelialization and diminished wound contraction *in vivo* [27]. An MCP-1 knockout was followed by delayed wound re-epithelialization and angiogenesis [29]. FGF7, also called keratinocyte growth factor (KGF), is mitogenic for keratinocytes and enhances the survival of epithelial cells in inflamed tissues [30]. HB-EGF stimulates keratinocyte proliferation [27]. TGF-β1 is released from platelets immediately after injury [31] and displays a chemoattractant activity for neutrophils, macrophages, and fibroblasts. A constant release of VEGF was observed in all 6 donors at 3 different time points after seeding (Fig. 5B). VEGF is one of the key regulators of angiogenesis and is mainly expressed by keratinocytes and macrophages. Its receptors (VEGFR-1 and -2) were detected in blood vessels of granulation tissue [27] suggesting a key role in wound healing. In contrast to VEGF, the constant release of the chemo-attractant molecule SDF-1α was detected only in one out of 6 donors (data not shown). However, as well as VEGF, it was found in protein extracts of ASC-filled sutures from all the donors (Fig. 5C). This result suggests that certain molecules accumulate within the suture acting locally at the wound edge or being further released while the suture is biodegraded. The healing potential of cytokines released by the filled sutures was clearly revealed in an *in vitro* wound-healing assay, where the open wound area was significantly decreased after applying conditioned media (Fig. 6). In agreement with previous publications [32], [33] our results suggest significant variations in the cytokine profile of different donors. This issue should be considered for *in vivo* applications to determine whether allogeneic or autologous cell transplantation is the most suitable therapeutic option. This is of particular interest for patients with impaired wound healing, where a treatment with allogeneic cells from healthy donors might be beneficial.

ASC-filled sutures and control sutures showed similarities regarding their elastic limit, but a significantly lower stiffness and maximum force. The significant decrease in the maximum force observed in the ASC-suture (Fig. 4B) could be partially explained by a mechanical disruption of the native structure of the suture during the ASC filling step or by a direct enzymatic degradation of the material by the seeded cells. We conclude that as long as the elastic limit of the ASC-filled sutures is not exceeded, the material can be used as a functionally enriched product. Reduced stiffness can also be beneficial if filled sutures are used within elastic tissues, in order to increase patient comfort and decrease tension at wound edges [34]. Experiments performed in an *ex vivo* wound model using human skin showed that ASCs survived and remained metabolically active after suturing (Fig. 7A). This shows that the filling of the sutures with ASCs is able to protect cells from shearing off while suturing, indicating that this approach is feasible in a clinical setting. In order to facilitate the clinical translation of this concept, we also showed that ASC-filled sutures could be cryopreserved (Fig. 7B). This issue is particularly relevant in approaches where ready to use sutures should be produced and stored for further clinical applications.

## Conclusion

This work demonstrates the feasibility of using ASC-filled sutures as a therapeutic approach for wound healing. We developed a storable and ready to use bioactive suture, which is feasible for a clinical setting and a production according to GMP standards is foreseeable. Our results clearly demonstrate that filled sutures release key molecules involved in angiogenesis, immunomodulation, and tissue remodeling. Here we extend the use of cell therapies in regenerative medicine, describing a new possible application. Further studies have to be performed to evaluate the healing effect of cell-filled sutures *in vivo*.
